# Adherence to individualized recall intervals for oral health examinations

**DOI:** 10.1002/cre2.686

**Published:** 2022-11-02

**Authors:** Anna Haukka, Minna Kaila, Jari Haukka, Anna M. Heikkinen

**Affiliations:** ^1^ Dental Care, Health Services, Social Services and Health Care City of Helsinki Finland; ^2^ Department of Public Health University of Helsinki Helsinki Finland; ^3^ Public Health Medicine, Department of Public Health University of Helsinki Helsinki Finland; ^4^ Department of Oral and Maxillofacial Diseases, Head and Neck Center University of Helsinki, Helsinki University Hospital Helsinki Finland; ^5^ Faculty of Medicine and Health Technology Tampere University Tampere Finland

**Keywords:** oral health, oral health examination, oral health services, recall interval

## Abstract

**Objectives:**

The aim of this follow‐up study was to investigate whether adults attend an oral health examination (OHE) based on their individual recall interval (IRI) without a reminder recall system.

**Methods:**

The study population included adults who were attending an OHE recommended by their dentists based on their IRI in public oral healthcare clinics of Helsinki City January 1, 2009−December 31, 2009. The inclusion criteria were as follows: alive until the end of IRI, length of the IRI of 12–60 months, and study participants had not been treated successfully by a dental specialist during the IRI period (*n* = 41,255). We used a multinomial model to identify the factors associated with the timing of OHE. The following predictors were included: oral health indices such as Decayed Teeth and the Community Periodontal Index, the length of the IRI based on an OHE in 2009, age, gender, socioeconomic status, presence of chronic diseases, and emergency appointment. Results were presented as odds ratios with 95% confidence intervals.

**Results:**

The OHE based on IRI occurred for 7505 individuals (18.2%) and the OHE was late for 9159 individuals (22.2%). A total of 24,591 (59.6%) adults did not undergo follow‐up OHE based on the IRI period of on time or late. Those who came on time for follow‐up OHE experienced less caries than those who came later. There was not much difference in periodontal health between the groups. The models indicated that having an emergency appointment was associated with a higher probability of having an OHE. A long IRI (37–60 months) was associated with a higher probability of not participating in OHE even late.

**Conclusions:**

It would be beneficial for patients to take appointments based on the recall interval. The results of this study indicated that more needs to be done to increase awareness in the adult population of the benefits and availability of follow‐up OHEs based on their IRI in oral healthcare.

## 1 INTRODUCTION

Oral health is an important part of an individual's health and well‐being. There are five driving factors that affect oral health: genetic and biological factors, the social environment, the physical environment, health behaviors, and access to healthcare services (Glick et al., 2016). Oral disease caries—untreated tooth decay—and especially periodontitis are widespread chronic diseases. However, both diseases and their progression are preventable with good oral hygiene practices and the ability to receive oral health services (OHS) at the right time (Selwitz et al., 2007; Tonetti et al., 2017). It is important that healthcare systems include OHS for the entire population (Kandelman et al., 2012) and provide access to oral health examinations (OHE) based on individually determined recall intervals (NICE National Institute for Health and Care Excellence, 2004; Patel et al., 2010).

The priority in oral health care is to perform more preventive rather than restorative treatments (Association, 2001). The recommendations on recall interval are based on evidence that regular attenders have better‐functioning teeth and are less likely to be suffering acute symptoms or require emergency treatments (Sheiham et al., 1985; Thomson et al., 2010). The randomized‐controlled trial of Clarkson et al. (2020) found no evidence of a difference in outcome between recall strategies on risk‐based recall interval, schedule 6 or 24 months for OHEs (Clarkson et al., 2020). The adjusted length of the risk‐based recall interval to follow‐up OHE was based on the National Institute for Health and Care Excellence guideline (NHS) (Clarkson et al., 2020; NICE National Institute for Health and Care Excellence, 2004). The results of the study carried out by Clarkson et al. (2020) confirmed that a variable risk‐based recall interval is not detrimental to oral health in terms of number of tooth surfaces with decay, gum disease, and quality of life (Clarkson et al., 2020).

In studies comparing oral health of regular and irregular attenders, the results of the Adult Dental Health Survey in the UK (year 1998) using the index of Decayed, Missing, Filling Teeth (DMFT) as an information of adult's oral health (≥16 years of age) provided evidence that former regular and irregular attenders had a significantly higher DMFT score than always regular attenders, although the number of filled teeth of irregular attenders was lower than regular attenders (Aldossary et al., 2015). Also, regular attenders had healthier periodontium than irregular attenders (Karimalakuzhiyil Alikutty & Bernabé, 2016). The results of other survey research have also been shown an association between regular dental attendance and better quality of life (Kaprio et al., 2012; Mc Grath & Bedi, 2001; Richards & Ameen, 2002; Torppa‐Saarinen et al., 2019).

The focus of individual recall is the continuing care regime (Clarkson et al., 2009) and appropriate timing for having an OHE. The individual recall interval (IRI) can be defined as the time period between the first and follow‐up OHE and IRI is based on the patient's needs (Mettes, 2005). Another significant aspect of OHE is based on the idea of a dual function as both a primary (prevent the occurrence of oral diseases) and a secondary (detected signs and symptoms of oral diseases) preventive measure (Riley et al., 2013). Therefore, recall intervals for follow‐up OHEs are intended to prevent caries and periodontal diseases (Patel et al., 2010) because caries lesions and especially periodontal infection can progress without an individual knowing. In our previous cross‐sectional study, it was shown that indices such as number of Decayed Teeth (DT), DMFT, the Community Periodontal Index (CPI), and the general health of individuals were associated with IRI (Haukka et al., 2020).

It is important that once IRI has been determined, a patient has access to follow‐up OHE. There are only a few studies that have considered how the IRI is adhered to among adults (Clarkson et al., 2020; Davenport et al., 2003; Woolfolk et al., 1999). The aim of our follow‐up, register‐based study was to explore how the IRI was implemented in our study population during 2009–2015 in public oral healthcare clinics in the city of Helsinki, Finland. We tried to identify factors associated with follow‐up OHEs that were based on the IRI. We hypothesized that those who had shorter IRIs attended the follow‐up OHE more often than those who had longer IRIs. We also investigated the factors predicting the follow‐up OHE.

## 2 METHODS

**Figure 1:**
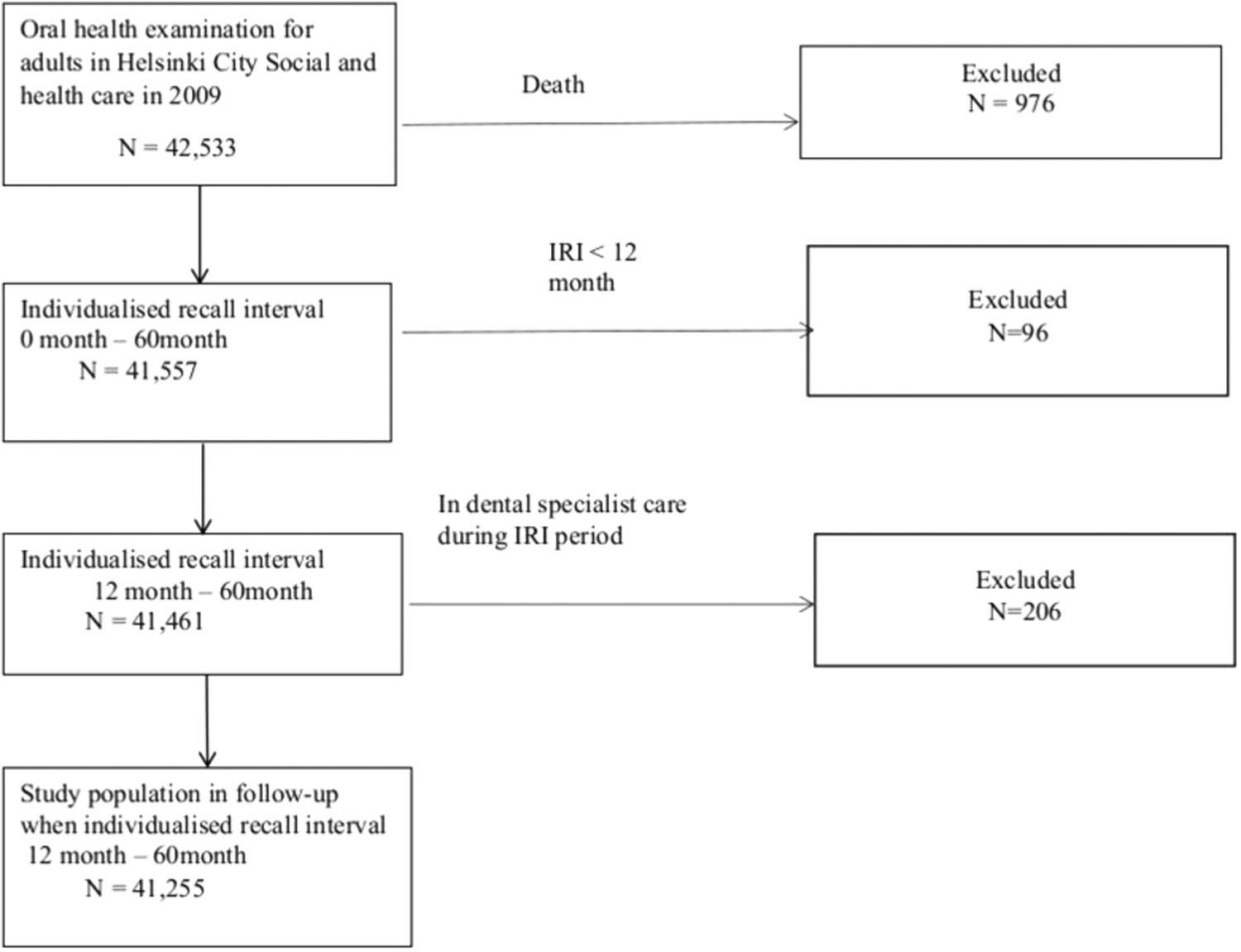
Study population flow chart. IRI, individual recall interval.

Our longitudinal study included all adults with IRIs determined by a dentist at an OHE in the public oral healthcare clinics of Helsinki City Social Services and Health Care from January 1, 2009−December 31, 2009. Information about OHEs was obtained from computerized medical records of visits in 2009. The IRI was between 0 and 60 months in 2009. Initially, the study source population comprised 42,533 adults (age range from 18 to 89 years). The socioeconomic status (SES) in the study population was very similar to that the general population of the city of Helsinki in 2000. The inclusion criteria were as follows: alive until the end of the IRI, the length of the IRI of 12–60 months, and study participants had not been treated successfully by a dental specialist during the IRI period. The resulting sample size was 41,255 adults (Figure 1). The follow‐up OHE was defined as visits when OHE included an assessment of all oral tissues, a diagnosis, a treatment plan, and assessment and IRI.

In Finland, data from different sources can be combined through the computerized register using unique Personal Identification numbers (PICs) (Gissler & Haukka, 2004). The information about SES was obtained from Statistics Finland and was categorized into eight categories: self‐employed or employers, upper‐level employees, lower‐level employees, manual workers, students, pensioners, unemployed, and unknown (Statistics Finland, n.d.). Information on a patient's chronic diseases was accessed from the special drug reimbursement register of the Finnish Social Insurance Institution (SII) (Overview of Benefit Programmes 2007, 2007). The following diseases were included: diabetes mellitus, Parkinson's disease and other comparable movement disorders, severe psychotic and other severe mental disorders, chronic cardiac insufficiency, disseminated connective tissue diseases, rheumatoid arthritis and comparable conditions, chronic asthma and similar chronic obstructive pulmonary diseases, chronic hypertension, chronic coronary heart disease and dyslipidemia associated with chronic coronary heart disease, and chronic arrythmias. Drug cost reimbursements are based on physician statements. The oral health medical records include special procedure codes for OHEs that are used in OHS. All codes are combined with payments of OHE, and treatments performed in public and as well as private oral health clinics. The codes have been provided by the board of the Finnish Institute for Health and Welfare.

The primary outcome of study was a categorical variable with tree levels: if a patient had attended an OHE by a dentist in the time window of 1 month before and 6 months after the IRI (OHE on time), the OHEs were performed from 7 to 24 months after the patient's IRI (OHE late) or not at all (no OHE) (Supporting Information: Figure S1). The process of accessing an OHE was the same from 2009 until the end of November 2015 (Haukka et al., 2020). The following predictor variables were available in both analyses: IRI length, age, gender, oral health indices on the date when OHE was carried out, SES, and information on chronic diseases. Oral health information that included indices concerning permanent teeth DT, DMFT, number of teeth, and CPI was recorded for the full mouth, including in the follow‐up OHE, and emergency appointment during the IRI period (yes/no). The IRI was defined to be categorical variable with categories 12 (reference category), 13–24, 25–36 and 37–60 months.

We modeled categorical outcome with a multinomial regression model using the category “OHE on time” as the reference level. Results are presented as odds ratios (OR) with 95% confidence intervals (CI). The above‐mentioned oral health and other variables were used as predictors in multinomial models.

The study was approved by the ethics committee of the Faculty of Medicine at the University of Helsinki (September 8, 2017 reference 09/2017). Permits to use register data were obtained from the City of Helsinki (January 5, 2018 reference 2017‐013665), Statistics Finland (January 3, 2019 reference TK‐52‐41‐19), and SII (January 31, 2019 reference 9/522/2019.

## 3 RESULTS

Our register‐based, follow‐up study investigated how well an OHE based on an IRI was adhered to after the index OHE in 2009. The population of the follow‐up study included 41,255 individuals (Table 1). The OHE based on the IRI was on time for 7505 individuals (18.2%) (Table 2). The number of individuals who had an OHE 7–24 months late was 9159 (22.2%) (Table 2). We observed no follow‐up OHE visit for 24,591 (59.6%) individuals (Table 1).

**Table 1:** Basic characteristics (demographics and oral health indicators) of the study population (*N* = 41,255)

The follow‐up oral health examination	OHE visit on time	OHE visit late	No OHE visit
*n*	7505	9159	24,591
age (mean [SD])	48.4 (16.6)	45.6 (16.2)	39.7 (15.5)
Gender (%)			
Male	2325 (31.0)	3165 (34.6)	9861 (40.1)
Female	5180 (69.0)	5994 (65.4)	14730 (59.9)
SES (%)			
Self‐employed or employers	150 (2.0)	214 (2.3)	760 (3.1)
Upper‐level employees	1188 (15.8)	1504 (16.4)	4645 (18.9)
Lower‐level employees	2078 (27.7)	2642 (28.8)	7273 (29.6)
Manual workers	778 (10.4)	1194 (13.0)	3708 (15.1)
Students	220 (2.9)	327 (3.6)	1407 (5.7)
Pensioners	2391 (31.9)	2178 (23.8)	3583 (14.6)
Unemployed	481 (6.4)	736 (8.0)	1972 (8.0)
Unknown	219 (2.9)	364 (4.0)	1243 (5.1)
Chronic diseases			
Diabetes (%)	366 (4.9)	370 (4.0)	659 (2.7)
Parkinson's disease (%)	13 (0.2)	22 (0.2)	32 (0.1)
Severe mental disorders (%)	295 (3.9)	318 (3.5)	576 (2.3)
Cardiac insufficiency (%)	39 (0.5)	51 (0.6)	83 (0.3)
Connective tissue diseases (%)	205 (2.7)	205 (2.2)	324 (1.3)
Asthma and similar obstructive pulmonary diseases (%)	398 (5.3)	431 (4.7)	798 (3.2)
Hypertension (%)	783 (10.4)	743 (8.1)	1132 (4.6)
Coronary heart disease (%)	267 (3.6)	254 (2.8)	406 (1.7)
Arrhythmias (%)	74 (1.0)	61 (0.7)	120 (0.5)
DMFT (mean [SD])†	18.0 (8.7)	17.0 (8.6)	13.5 (9.0)
DT (mean [SD])‡	1.1 (2.0)	1.6 (2.5)	1.8 (2.9)
Number of teeth (mean [SD])	26.8 (5.1)	27.3 (4.9)	28.0 (5.22)
CPI (max) (%) §			
0	376 (5.0)	393 (4.3)	1202 (4.9)
1	520 (6.9)	618 (6.7)	1560 (6.3)
2	4723 (62.9)	5988 (65.4)	16819 (68.4)
3	1480 (19.7)	1662 (18.1)	3809 (15.5)
4	385 (5.1)	478 (5.2)	1040 (4.2)
X (edentulous)	21 (0.3)	20 (0.2)	161 (0.7)
Individual recall interval (IRI) months (%)			
12	806 (10.7)	1009 (11.0)	2332 (9.5)
13–24	4274 (56.9)	5533 (60.4)	13314 (54.1)
25–36	2132 (28.4)	2308 (25.2)	7395 (30.1)
37–60	293 (3.9)	309 (3.4)	1550 (6.3)
Emergency appointment (%)	3765 (50.2)	4897 (53.5)	7471 (30.4)

*Note*: For continuous variables, means (standard deviations) are presented and for categorical variables, frequencies (%) are presented.

Abbreviations: Chronic diseases, health information based on entitlement to the Drug Reimbursement Register of Finnish Social Insurance Institution; CPI, Community Periodontal Index (CPI for the maximum value of individual) (baseline) in OHE year 2009 for those who had OHE based on IRI, after 7–24 months, or no OHE visit: 0 = healthy, 1 = bleeding on probing, 2 = calculus, 3 = pocket depth 4–5 mm, 4 = pocket depth 6 mm or more; DMFT, decayed, missing, filled teeth (baseline) in OHE year 2009 for those who had OHE based on IRI, after 7–24 months, or no OHE visit; DT, decayed teeth (baseline) in OHE year 2009 for those who had OHE based on IRI, after 7–24 months, or no OHE visit; Emergency appointment, need emergency treatment because of oral health dysfunction, pain, or infection during IRI; Number of teeth, (wisdom teeth included) (baseline) in OHE year 2009 for those who had OHE based on IRI, after 7–24 months, or no OHE visit; Late visit, between 7 and 24 months after IRI; SES, Socioeconomic status (Statistics Finland); Visit on time, 1 month before or 6 months after IRI.

**Table 2:** Oral health indicators on follow‐up oral health examination (OHE) of the study population (*N* = 41,255)

The follow‐up oral health examination	OHE visit on time	OHE visit late
*n*	7505	9159
DMFT (fu) (mean [SD])†	18.5 (8.5)	17.6 (8.3)
DT (fu) (mean [SD])‡	1.0 (1.8)	1.4 (2.3)
DT (fu) (%) 0	3889 (51.8)	4195 (45.8)
DT (fu) (%) 1–3	2566 (34.2)	3294 (36.0)
DT (fu) (%) >3	519 (6.9)	1039 (11.3)
Missing (%)	531 (7.1)	631 (6.9)
Number of teeth fu (mean [SD])	26.5 (5.2)	26.8 (5.1)
CPI (max) (fu) (%) §		
0	289 (4.0)	333 (3.8)
1	569 (7.8)	624 (7.1)
2	4366 (60.0)	5554 (63.1)
3	1610 (22.1)	1807 (20.5)
4	426 (5.9)	457 (5.2)
X (edentulous)	19 (0.3)	23 (0.3)
Missing (%)	226 (2.0)	361 (3.9)

*Note*: For continuous variables, means (standard deviations) are presented and for categorical variables, frequencies (%) are presented. Note that indicators are not available for individuals without follow‐up OHE (*N* = 24,591).

Abbreviations: Chronic diseases, health information based on entitlement to the Drug Reimbursement Register of Finnish Social Insurance Institution; CPI (fu), Community Periodontal Index (CPI for the maximum value of individual) in follow‐up OHE based on IRI or after 7–24 months; DMFT (fu), decayed, missing, filled teeth in follow‐up OHE based on IRI or after 7–24 months; DT (fu), decayed teeth in follow‐up OHE based on IRI or after 7–24 months; Late visit, between 7 and 24 months after IRI; Number of teeth fu, (wisdom teeth included) in follow‐up OHE based on IRI or after 7–24 months; SES, Socioeconomic status (Statistics Finland); Visit on time, 1 month before or 6 months after IRI.

Factors that influenced follow‐up OHE were oral health, IRI, gender, SES, and general health. The majority who attended on time to OHE were women, and the largest SES group included pensioners. Individuals who had chronic diseases were more likely to have an OHE according to their IRI or late (Table 1). When evaluating oral health index DT, we observed that those who followed their IRI had less caries than those who did not (Table 2). However, on comparison of the maximum value of CPI, there were no substantial differences between groups that were on time or late (Table 2).

**Figure 2:**
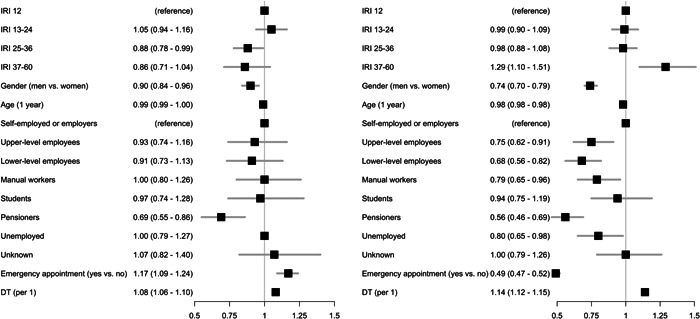
Odds ratios (ORs) and 95% confidence intervals for attending follow‐up oral health examination. The outcome variable was timing of oral health examination (OHE) late on the left and no on the right. On time was used as a reference. ORs modeled using combinations of individual recall interval (IRI), gender, age, socioeconomic status (SES), emergency appointment (emg.), and number of decayed teeth (DT) as predictors.

The results of the multinomial model show ORs for categories “OHE visit late” and “no OHE visit” compared to “OHE visit on time” (Table 3). For example, the second row of Table 3 indicates that the OR for “OHE visit late” compared to “OHE visit on time” is 0.93 (95% CI: 0.93–1.14) when we compare IRI 13–24 months to IRI 12 months, and OR for “No OHE visit” compared to “OHE visit on time” is 1.08 (0.99–1.17). ORs higher than unity are interpreted to indicate that the probability of being in a category is increased and vice versa. Based on modeling, we found that there was an association between the length of IRI and the timing of follow‐up OHE (Table 3). The model without covariates indicated that the longest IRI (37–60 months) was associated with a higher probability of not attending follow‐up OHE. However, with some combinations of covariates such as gender, age, and SES, this association was not detected. On the other hand, when the DT index was included as a covariate, the longest IRI was associated with a high probability of not attending (Figure 2, Supporting Information: Table S1).

**Table 3:** Results of multinomial models for the probability of achieved individual recall interval (IRI). The outcome variable was the timing of oral health examination (OHE) (on time, late, no)

	OHE visit late OR (95% CI)	No OHE visit OR (95% CI)
IRI 12 (no variables)	(reference)	(reference)
IRI 13–24 (no variables)	1.03 (0.93−1.14)	1.08 (0.99–1.17)
IRI 25–36 (no variables)	0.86 (0.77−0.97)	1.20 (1.09–1.32)
IRI 37–60 (no variables)	0.84 (0.70−1.01)	1.83 (1.58–2.12)
Multinomial model		
IRI 12 (age and gender)	(reference)	(reference)
IRI 13–24 (age and gender)	0.96 (0.86–1.06)	0.88 (0.80–0.96)
IRI 25–36 (age and gender)	0.76 (0.68–0.85)	0.85 (0.77–0.94)
IRI 37–60 (age and gender)	0.70 (0.58–0.84)	1.12 (0.96–1.31)
Multinomial model		
IRI 12 (age, gender, SES)	(reference)	(reference)
IRI 13–24 (age, gender, SES)	0.94 (0.85–1.04)	0.87 (0.79–0.95)
IRI 25–36 (age, gender, SES)	0.74 (0.66–0.83)	0.84 (0.76–0.93)
IRI 37–60 (age, gender, SES)	0.69 (0.57–0.84)	1.12 (0.96–1.31)
Multinomial model		
IRI 12 (age, gender, SES, chronic diseases)	(reference)	(reference)
IRI 13–24 (age, gender, SES, chronic diseases)	0.94 (0.84–1.04)	0.86 (0.78–0.94)
IRI 25–36 (age, gender, SES, chronic diseases)	0.74 (0.66–0.83)	0.82 (0.75–0.91)
IRI 37–60 (age, gender, SES, chronic diseases)	0.69 (0.57–0.83)	1.10 (0.94–1.28)
Multinomial model		
IRI 12 (age, gender, SES, number of teeth)	(reference)	(reference)
IRI 13–24 (age, gender, SES, number of teeth)	0.95 (0.85–1.05)	0.90 (0.82–0.99)
IRI 25–36 (age, gender, SES, number of teeth)	0.67 (0.67–0.84)	0.88 (0.80–0.97)
IRI 37–60 (age, gender, SES, number of teeth)	0.58 (0.58–0.84)	1.18 (1.01–1.37)
Multinomial model		
IRI 12 (age, gender, SES, CPI)	(reference)	(reference)
IRI 13–24 (age, gender, SES, CPI)	0.94 (0.85–1.04)	0.88 (0.81–0.97)
IRI 25–36 (age, gender, SES, CPI)	0.74 (0.66–0.83)	0.85 (0.77–0.94)
IRI 37–60 (age, gender, SES, CPI)	0.69 (0.57–0.83)	1.13 (0.96–1.31)
Multinomial model		
IRI 12 (age, gender, SES, DT)	(reference)	(reference)
IRI 13–24 (age, gender, SES, DT)	1.04 (0.93–1.15)	1.03 (0.94–1.13)
IRI 25–36 (age, gender, SES, DT)	0.86 (0.76–0.96)	1.07 (0.97–1.19)
IRI 37–60 (age, gender, SES, DT)	0.83 (0.68–1.00)	1.51 (1.29–1.77)
Multinomial model		
IRI 12 (age, gender, SES, DT, emg.)	(reference)	(reference)
IRI 13–24 (age, gender, SES, DT, emg.)	1.05 (0.94−1.16)	0.99 (0.90−1.09)
IRI 25–36 (age, gender, SES, DT, emg.)	0.88 (0.78−0.99)	0.98 (0.88−1.08)
IRI 37–60 (age, gender, SES, DT, emg.)	0.86 (0.71−1.04)	1.29 (1.10−1.51)

*Note*: On time was used as a reference. Odds ratios (ORs) modeled using combinations of age, gender, socioeconomic status (SES), chronic diseases, number of teeth, maximum value of Community Periodontal Index (CPI), number of decayed teeth (DT), and DT combined with emergency appointment (emg.) as predictors.

The importance of earlier contact via emergency appointments was observed in models, where we adjusted with length of IRI, age, gender, SES, and emergency appointment (Figure 2). Earlier contact via an emergency appointment was associated with a positive effect on attending follow‐up OHE. The OR for emergency visit was 0.49 (0.47–0.52) for not attending follow‐up (Figure 2, Supporting Information: Table S1). In other words, having emergency visits increased the probability of follow‐up OHE.

## 4 DISCUSSION

The findings of our follow‐up study showed that in real life, only 18.2% of the study population adhered to the possibility to book the appointment for a follow‐up OHE appointment based on their IRI, and 22.0% of the study population had follow‐up OHE late. The rest of the study population, 59.6%, did not attend follow‐up OHE based on IRI or attended late. According to multinomial models, the combination of covariates included in the models modified the association between IRI and the timing of attending OHE. In our opinion, including the DT index as a covariate is reasonable because it is a confounding variable that has an effect on both IRI and the timing of OHE. Thus, we consider models including above mentioned variables the most interpretable, and used them in our inference. Results (Table 3) show that there was no difference if OHE was on time or late according to IRI. On the other hand, longest IRI (37–60 months) was associated with a higher probability of not attending follow‐up OHE when DT was taken into account. There was no reminder recall system for booking the next appointment, which could be considered as a barrier to adhering to the IRI.

The evidence of optimal recall time for individual has been evaluated in systematic reviews (Davenport et al., 2003; Fee et al., 2020; Riley et al., 2013). The results of an RCT study showed that there is no adverse effect of variable risk‐based recall interval on oral health; the maximum length of the risk‐based recall interval was based on the guidelines of NHS (Clarkson et al., 2020; Fee et al., 2020). In our study, the longest recall interval was 37–60 months. The study models showed that very long IRI (37–60 months) had an association with a higher probability of not attending follow‐up OHE. Too long a time scale of IRI could be a reason for the decrease in regular attendance in adults.

Generally, access to OHEs is an opportunity to provide motivational and preventive support to change behaviors in individuals and IRI is one model that provides support to adults to access OHEs on time. In our study, we found that the prevalence of periodontal disease in adults did not decrease during the IRI period. The result was similar to those obtained from the cross‐sectional Health 2000 and 2011 surveys of Finnish adults (Seppo et al., 2012; Suominen‐Taipale et al., 2008). The finding of no improvement in CPI in the follow‐up OHE is an important topic for future research to explore; periodontitis, in the early stages, usually progresses without symptoms and we need to find ways to improve the periodontal health of adults in the future. Furthermore, research findings have shown that irregular attenders may simultaneously have a higher prevalence of caries and more severe periodontitis (Durand et al., 2019; P. T. Mattila et al., 2010). The findings of our follow‐up study are in accordance with those of a previous study that showed that patients who had an OHE on time based on their IRI had fewer caries than their first visit (Clarkson & Worthington, 1993). There was also a reduction of DT if the OHE occurred late.

Our study confirmed findings of other studies reporting that regular attenders were women more often than men (Suominen‐Taipale et al., 2008; Woolfolk et al., 1999). In contrast to earlier findings, however, in our study, pensioners and lower‐level employees were the ones most likely to come on time to OHEs (Suominen‐Taipale et al., 2008; Thomson et al., 2000; Woolfolk et al., 1999). The legislation of the Finnish Public Oral Health Care (POHC) based on regulation was issued on December 1, 2002 including an article that people of all ages are allowed to access POHC (*FINLEX ®—Säädökset alkuperäisinä*, n.d.). This could be one reason why the majority of pensioners had on‐time OHEs.

Nevertheless, there is the need to develop strategies that can reach those adults who do not make appointments for OHEs (Clarkson et al., 2020). It has been suggested that a recall system could be useful and could encourage patients to make an appointment. The importance of IRI and a recall system is based on several aspects. First of all, the link between some general diseases and oral health should not be neglected when the dentist is determining the IRI (Haukka et al., 2020). Undiagnosed diabetes or suboptimal diabetes, as well as prediabetes, rheumatoid arthritis, and possibly hyposalivation are risk factors for development of oral diseases (Chapple et al., 2017) (Genco & Borgnakke, 2013; Tonetti et al., 2015). Also, associations between periodontitis and cardiovascular diseases (Chistiakov et al., 2016; K. J. Mattila et al., 2005; Pussinen et al., 2019), hypertension (Genco & Borgnakke, 2013; Tonetti et al., 2017), and Alzheimer's disease have been reported (Kamer et al., 2008). Results from earlier studies showed that regular attendance could even fell by age (Åstrøm et al., 2014; Thomson et al., 2010). Above all, recall practice could improve the equal utilization of OHS by adults (Nguyen et al., 2005).

In our study, models confirmed the importance of reminders because the attendance was higher if there was an emergency appointment before the follow‐up OHE. The results showed that 16,133 (39.1%) individuals required an emergency appointment within their IRI period. It is of note that the follow‐up OHE was observed for 53.7% of individuals with emergency appointment. There have been discussions that the positive effect of routine attendance on oral health might be due to a “healthy user effect” (Thomson et al., 2010). In our study, the IRI was based on an individual's oral health status and possible systematic disorders (Haukka et al., 2020) and there was a benefit for adults who attend an OHE based on their IRI.

Some limitations related to the data should be taken into account. There were 24,519 (59.6%) individuals who did not attend the follow‐up OHE based on their IRI or later. It is improbable that migration from Helsinki could be the reason because, from 2009 to 2015, the migration rate was only 5.4%. Our data were from registers only and there was no information about the reasons for not attending OHE. The strength of this study was that our follow‐up study included oral health information on 41,255 adults who attended OHEs, and dentists had determined IRIs in the POHC of Helsinki in 2009. Our data were extensive and population‐based, and represent the source population of Helsinki City well. The distribution of SES in the study population in 2009 was very similar to that of the general population of the city of Helsinki in 2000 (Haukka et al., 2020).

Our result revealed that without an appropriate reminder recall system, follow‐up OHE seldom occurs, and opportunities for preventive oral health care may be lost. Oral health was better for those who had IRIs between 37 and 60 months in 2009 (Haukka et al., 2020). Our study shows that without a recall system for those who had the longest IRI, it was probably hard to remember to book the follow‐up OHE.

In conclusion, the IRI is a useful procedure to utilize OHS systematically if it is based on a patient's treatment needs. The awareness of severe periodontitis risk should be considered to support access for adults to OHS. We emphasise that dentists should inform patients about the benefits of regular visits to OHE based on IRIs. Our population‐based follow‐up study shows that there is a need to support adults and allow easier access to book appointments to OHE based on IRIs. The organizations and administrations of POHC should resolve the question of how to inform adults about follow‐up OHEs. Digital facilities could provide solutions to these issues.

## AUTHOR CONTRIBUTIONS

All the authors have made substantial contributions: Anna Haukka, Minna Kaila, Jari Haukka, and Anna M. Heikkinen were involved in conceptualization and design of the study; Anna Haukka and Jari Haukka were involved in data acquisition and analysis; and Anna Haukka, Minna Kaila, Jari Haukka, and Anna M. Heikkinen were involved in reporting and interpretation of the results of the study. All the authors were involved in drafting the article or revising it critically for important intellectual content, and all have approved the final version.

## CONFLICT OF INTEREST

The authors declare no conflict of interest.

## ETHICS STATEMENT

The Ethics Committee of the Faculty of Medicine at the University of Helsinki reviewed and approved our study design and data collection methods (reference 09/2017). Patient consent for publication was not required because deidentified data were used.

## Supporting information

Supporting information.

Supporting information.

Supporting information.

## Data Availability

The data that support the findings of this study are available from Statistics Finland, the Social Insurance Institution, Social Services and Health Care, City of Helsinki, but restrictions apply to the availability of these data, which were used under license for the current study, and so are not publicly available. Data are, however, available from the authors upon reasonable request and with the permission of Statistics Finland, the Social Insurance Institution, Social Services and Health Care, City of Helsinki.

## References

[cre2686-bib-0001] Aldossary, A. , Harrison, V. E. , & Bernabé, E. (2015). Long‐term patterns of dental attendance and caries experience among British adults: A retrospective analysis. European Journal of Oral Sciences, 123(1), 39–45. 10.1111/eos.12161 25521216

[cre2686-bib-0002] Association, A. D. E. (2001). Diagnosis and management of dental caries throughout life. National Institutes of Health Consensus Development Conference statement, March 26–28, 2001. Journal of Dental Education, 65(10), 1162–1168. 11699994

[cre2686-bib-0003] Åstrøm, A. N. , Ekback, G. , Ordell, S. , & Nasir, E. (2014). Long‐term routine dental attendance: Influence on tooth loss and oral health‐related quality of life in Swedish older adults. Community Dentistry and Oral Epidemiology, 42(5), 460–469. 10.1111/cdoe.12105 24712734

[cre2686-bib-0004] Chapple, I. L. C. , Bouchard, P. , Cagetti, M. G. , Campus, G. , Carra, M. ‐C. , Cocco, F. , Nibali, L. , Hujoel, P. , Laine, M. L. , Lingström, P. , Manton, D. J. , Montero, E. , Pitts, N. , Rangé, H. , Schlueter, N. , Teughels, W. , Twetman, S. , Van Loveren, C. , Van der Weijden, F. , … Schulte, A. G. (2017). Interaction of lifestyle, behaviour or systemic diseases with dental caries and periodontal diseases: Consensus report of group 2 of the joint EFP/ORCA workshop on the boundaries between caries and periodontal diseases. Journal of Clinical Periodontology, 44, S39–S51. 10.1111/jcpe.12685 28266114

[cre2686-bib-0005] Chistiakov, D. A. , Orekhov, A. N. , & Bobryshev, Y. V. (2016). Links between atherosclerotic and periodontal disease. Experimental and Molecular Pathology, 100(1), 220–235. 10.1016/j.yexmp.2016.01.006 26777261

[cre2686-bib-0006] Clarkson, J. E. , Amaechi, B. T. , Ngo, H. , & Bonetti, D. (2009). Recall, Reassessment and Monitoring. In N. Pitts (Ed.), Monographs in oral science (Vol. 21, pp. 188–198). KARGER. 10.1159/000224223 19494686

[cre2686-bib-0007] Clarkson, J. E. , Pitts, N. B. , Goulao, B. , Boyers, D. , Ramsay, C. R. , Floate, R. , Braid, H. J. , Fee, P. A. , Ord, F. S. , Worthington, H. V. , van der Pol, M. , Young, L. , Freeman, R. , Gouick, J. , Humphris, G. M. , Mitchell, F. E. , McDonald, A. M. , Norrie, J. D. , Sim, K. , … Ricketts, D. (2020). Risk‐based, 6‐monthly and 24‐monthly dental check‐ups for adults: The INTERVAL three‐arm RCT. Health Technology Assessment, 24(60), 1–138. 10.3310/hta24600 PMC7701991 33215986

[cre2686-bib-0008] Clarkson, J. E. , & Worthington, H. V. (1993). Association between untreated caries and age, gender and dental attendance in adults. Community Dentistry and Oral Epidemiology, 21(3), 126–128. 10.1111/j.1600-0528.1993.tb00735.x 8348783

[cre2686-bib-0009] Davenport, C. F. , Elley, K. M. , Fry‐Smith, A. , Taylor‐Weetman, C. L. , & Taylor, R. S. (2003). The effectiveness of routine dental checks: A systematic review of the evidence base. British Dental Journal, 195(2), 87–98. 10.1038/sj.bdj.4810337 12881749

[cre2686-bib-0010] Durand, R. , Roufegarinejad, A. , Chandad, F. , Rompré, P. H. , Voyer, R. , Michalowicz, B. S. , & Emami, E. (2019). Dental caries are positively associated with periodontal disease severity. Clinical Oral Investigations, 23, 3811–3819. 10.1007/s00784-019-02810-6 30693397

[cre2686-bib-0011] Fee, P. A. , Riley, P. , Worthington, H. V. , Clarkson, J. E. , Boyers, D. , & Beirne, P. V. (2020). Recall intervals for oral health in primary care patients. The Cochrane Database of Systematic Reviews, 10(10), 004346. 10.1002/14651858.CD004346.pub5 PMC8256238 33053198

[cre2686-bib-0012] FINLEX® ‐ Säädökset alkuperäisinä: Laki kansanterveyslain 14 ja 49 §:n muuttamisesta 1219/2000 . (n.d.). Retrieved 9 June 2019, from https://www.finlex.fi/fi/laki/alkup/2000/20001219

[cre2686-bib-0013] Genco, R. J. , & Borgnakke, W. S. (2013). Risk factors for periodontal disease. Periodontology 2000, 62(1), 59–94. 10.1111/j.1600-0757.2012.00457.x 23574464

[cre2686-bib-0014] Gissler, M. , & Haukka, J. (2004). Finnish health and social welfare registers in epidemiological research. Norsk Epidemiologia, 2004(14), 113–120.

[cre2686-bib-0015] Glick, M. , Williams, D. M. , Kleinman, D. V. , Vujicic, M. , Watt, R. G. , & Weyant, R. J. (2016). A new definition for oral health developed by the FDI World Dental Federation opens the door to a universal definition of oral health. The Journal of the American Dental Association, 147(12), 915–917. 10.1016/j.adaj.2016.10.001 27886668

[cre2686-bib-0016] Haukka, A. , Heikkinen, A. M. , Haukka, J. , & Kaila, M. (2020). Oral health indices predict individualised recall interval. Clinical and Experimental Dental Research, 6, 585–595. 10.1002/cre2.319 32776480 PMC7745075

[cre2686-bib-0017] Kamer, A. R. , Craig, R. G. , Dasanayake, A. P. , Brys, M. , Glodzik‐Sobanska, L. , & de Leon, M. J. (2008). Inflammation and Alzheimer's disease: Possible role of periodontal diseases. Alzheimer's & Dementia, 4(4), 242–250. 10.1016/j.jalz.2007.08.004 18631974

[cre2686-bib-0018] Kandelman, D. , Arpin, S. , Baez, R. J. , Baehni, P. C. , & Petersen, P. E. (2012). Oral health care systems in developing and developed countries. Periodontology 2000, 60(1), 98–109. 10.1111/j.1600-0757.2011.00427.x 22909109

[cre2686-bib-0019] Kaprio, H. , Suominen, A. L. , & Lahti, S. (2012). Association between subjective oral health and regularity of service use. European Journal of Oral Sciences, 120(3), 212–217. 10.1111/j.1600-0722.2012.00955.x 22607337

[cre2686-bib-0020] Karimalakuzhiyil Alikutty, F. , & Bernabé, E. (2016). Long‐term regular dental attendance and periodontal disease in the 1998 adult dental health survey. Journal of Clinical Periodontology, 43(2), 114–120. 10.1111/jcpe.12496 26932321

[cre2686-bib-0021] Mattila, K. J. , Pussinen, P. J. , & Paju, S. (2005). Dental infections and cardiovascular diseases: A review. Journal of Periodontology, 76(11S), 2085–2088. 10.1902/jop.2005.76.11-S.2085 29539055

[cre2686-bib-0022] Mattila, P. T. , Niskanen, M. C. , Vehkalahti, M. M. , Nordblad, A. , & Knuuttila, M. L. E. (2010). Prevalence and simultaneous occurrence of periodontitis and dental caries. Journal of Clinical Periodontology, 37(11), 962–967. 10.1111/j.1600-051X.2010.01620.x 20958340

[cre2686-bib-0023] McGrath, C. , & Bedi, R. (2001). Can dental attendance improve quality of life? British Dental Journal, 190(5), 262–265. 11303687 10.1038/sj.bdj.4800944

[cre2686-bib-0024] Mettes, D. (2005). Insufficient evidence to support or refute the need for 6‐monthly dental check‐ups. What is the optimal recall frequency between dental checks? Evidence‐Based Dentistry, 6(3), 62–63. 16184154 10.1038/sj.ebd.6400341

[cre2686-bib-0025] Nguyen, L. , Häkkinen, U. , & Rosenqvist, G. (2005). Determinants of dental service utilization among adults—The case of Finland. Health Care Management Science, 8(4), 335–345. 16379416 10.1007/s10729-005-4143-7

[cre2686-bib-0026] NICE National Institute for Health and Care Excellence . (2004). Dental recall | Recall interval between routine dental examination. NICE. https://www.nice.org.uk/guidance/cg19

[cre2686-bib-0027] Overview of Benefit Programmes 2007 . (2007). In *Statistical Yearbook of Social Insurance* , Institution, Finland (pp. 332–370). Social Insurance Institution, Official Statistics of Finland.

[cre2686-bib-0028] Patel, S. , Bay, R. C. , & Glick, M. (2010). A systematic review of dental recall intervals and incidence of dental caries. The Journal of the American Dental Association, 141(5), 527–539. 10.14219/jada.archive.2010.0225 20436100

[cre2686-bib-0029] Pussinen, P. J. , Paju, S. , Koponen, J. , Viikari, J. S. A. , Taittonen, L. , Laitinen, T. , Burgner, D. P. , Kähönen, M. , Hutri‐Kähönen, N. , Raitakari, O. T. , & Juonala, M. (2019). Association of childhood oral infections with cardiovascular risk factors and subclinical atherosclerosis in adulthood. JAMA Network Open, 2(4), e192523. 10.1001/jamanetworkopen.2019.2523 31026022 PMC6487573

[cre2686-bib-0030] Richards, W. , & Ameen, J. (2002). The impact of attendance patterns on oral health in a general dental practice. British Dental Journal, 193(12), 697–702. 10.1038/sj.bdj.4801664 12529726

[cre2686-bib-0031] Riley, P. , Worthington, H. V. , Clarkson, J. E. , & Beirne, P. V. (2013). Recall intervals for oral health in primary care patients. Cochrane Database of Systematic Reviews, 12, 33. 10.1002/14651858.CD004346.pub4 24353242

[cre2686-bib-0032] Selwitz, R. H. , Ismail, A. I. , & Pitts, N. B. (2007). Dental caries. The Lancet, 369(9555), 51–59. 10.1016/S0140-6736(07)60031-2 17208642

[cre2686-bib-0033] Seppo, K. , Annamari, L. , & Noora (toim.), R . (2012). Terveys, toimintakyky ja hyvinvointi Suomessa 2011 (No. 68). Terveyden ja hyvinvoinnin laitos.

[cre2686-bib-0034] Sheiham, A. , Maizels, J. , Cushing, A. , & Holmes, J. (1985). Dental attendance and dental status. Community Dentistry and Oral Epidemiology, 13(6), 304–309. 10.1111/j.1600-0528.1985.tb00461.x 3866648

[cre2686-bib-0035] Statistics Finland . (n.d.). Classification of Socio‐economic Groups. Statistics Finland. Retrieved 18 June 2020, from https://www.stat.fi/en/luokitukset/sosioekon_asema/

[cre2686-bib-0036] Suominen‐Taipale, A. L. , Nordblad, A. , Vehkalahti, M. & Aromaa, A. (Ed.). (2008). Oral health in the Finnish adult population, Health 2000 Survey. Publications of the National Public Health Institute.

[cre2686-bib-0037] Thomson, W. M. , Poulton, R. , Kruger, E. , & Boyd, D. (2000). Socio–economic and behavioural risk factors for tooth loss from age 18 to 26 among participants in the Dunedin Multidisciplinary Health and development study. Caries Research, 34(5), 361–366. 10.1159/000016610 11014902

[cre2686-bib-0038] Thomson, W. M. , Williams, S. M. , Broadbent, J. M. , Poulton, R. , & Locker, D. (2010). Long‐term dental visiting patterns and adult oral health. Journal of Dental Research, 89(3), 307–311. 10.1177/0022034509356779 20093674 PMC2821461

[cre2686-bib-0039] Tonetti, M. S. , Eickholz, P. , Loos, B. G. , Papapanou, P. , van der Velden, U. , Armitage, G. , Bouchard, P. , Deinzer, R. , Dietrich, T. , Hughes, F. , Kocher, T. , Lang, N. P. , Lopez, R. , Needleman, I. , Newton, T. , Nibali, L. , Pretzl, B. , Ramseier, C. , Sanz‐Sanchez, I. , … Suvan, J. E. (2015). Principles in prevention of periodontal diseases. Journal of Clinical Periodontology, 42, S5–S11. 10.1111/jcpe.12368 25639948

[cre2686-bib-0040] Tonetti, M. S. , Jepsen, S. , Jin, L. , & Otomo‐Corgel, J. (2017). Impact of the global burden of periodontal diseases on health, nutrition and wellbeing of mankind: A call for global action. Journal of Clinical Periodontology, 44(5), 456–462. 10.1111/jcpe.12732 28419559

[cre2686-bib-0041] Torppa‐Saarinen, E. , Suominen, A. L. , Lahti, S. , & Tolvanen, M. (2019). Longitudinal pathways between perceived oral health and regular service use of adult Finns. Community Dentistry and Oral Epidemiology, 47(5), 374–380. 10.1111/cdoe.12478 31179572

[cre2686-bib-0042] Woolfolk, M. W. , Lang, W. P. , Borgnakke, W. S. , Taylor, G. W. , Ronis, D. L. , & Nyquist, L. V. (1999). Determining dental checkup frequency. The Journal of the American Dental Association, 130(5), 715–723. 10.14219/jada.archive.1999.0282 10332137

