# Supplementary figures and images for: Adherence to individualized recall intervals for oral health examinations
Source: Clin Exp Dent Res. 2022 Nov 2;9(1):177–85. doi: 10.1002/cre2.686 (PMC9932253; doi:10.1002/cre2.686)

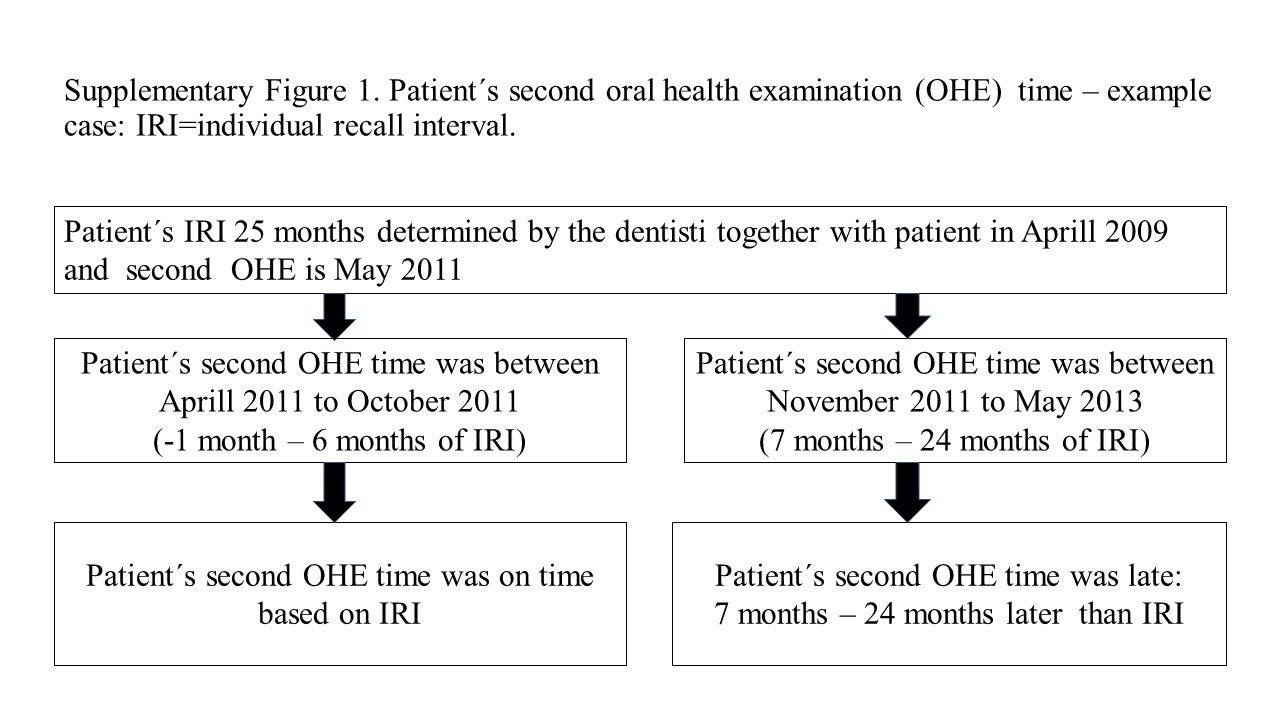

Supplement: Supplementary file 1 — Supporting information. [file CRE2-9-177-s001.jpg]
