# Supplementary material for: Adherence to individualized recall intervals for oral health examinations
Source: Clin Exp Dent Res. 2022 Nov 2;9(1):177–85. doi: 10.1002/cre2.686 (PMC9932253; doi:10.1002/cre2.686)
Supplement: Supplementary file 3 — Supporting information. [file CRE2-9-177-s003.docx]

Supplementary Table 1. Results of multinomial models for probability of achieved individual recall interval (IRI). Outcome variable was timing of oral health examination (OHE) (on-time, late, no). On-time was used as reference. Odds ratios (OR) modelled using combinations of age, gender, socioeconomic status (SES), emergency appointment (emg), maximum value of Community Periodontal Index (CPI), and number of decayed teeth (DT) as predictors.

|  | **OHE visit late**  **OR (95% CI)** | **No OHE visit**  **OR (95% CI)** |
| --- | --- | --- |
| IRI 12 (no variables) | (reference) | (reference) |
| IRI 13–24 (no variables) | 1.03 (0.93 - 1.14) | 1.08 (0.99 – 1.17) |
| IRI 25–36 (no variables) | 0.86 (0.77 - 0.97) | 1.20 (1.09 – 1.32) |
| IRI 37–60 (no variables) | 0.84 (0.70 - 1.01) | 1.83 (1.58 – 2.12) |
| Multinomial model |  |  |
| IRI 12 (age and gender) | (reference) | (reference) |
| IRI 13-24 (age and gender) | 0.96 (0.86 – 1.06) | 0.88 (0.80 – 0.96) |
| IRI 25-36 (age and gender) | 0.76 (0.68 – 0.85) | 0.85 (0.77 – 0.94) |
| IRI 37-60(age and gender) | 0.70 (0.58 – 0.84) | 1.12 (0.96 – 1.31) |
| Gender: woman | 0.85 (0.80 – 0.91) | 0.66 (0.62 – 0.70) |
| Age | 0.99 (0.99 – 0.99) | 0.97 (0.97 – 0.97) |
| Multinomial model |  |  |
| IRI 12 (age, gender, SES) | (reference) | (reference) |
| IRI 13-24 (age, gender, SES) | 0.94 (0.85 – 1.04) | 0.87 (0.79 – 0.95) |
| IRI 25-36 (age, gender, SES) | 0.74 (0.66 – 0.83) | 0.84 (0.76 – 0.93) |
| IRI 37-60 (age, gender, SES) | 0.69 (0.57 – 0.84) | 1.12 (0.96 – 1.31) |
| Gender: woman | 0.86 (0.81 – 0.92) | 0.68 (0.64 – 0.72) |
| Age | 0.99 (0.99 – 1.00) | 0.97 (0.97 – 0.97) |
| SES: |  |  |
| Upper-level employees | 0.91 (0.73 – 1.14) | 0.77 (0.64 – 0.93) |
| Lower-level employees | 0.91 (0.74 – 1.14) | 0.69 (0.58 – 0.83) |
| Manual workers | 1.04 (0.83 – 1.31) | 0.82 (0.68 – 1.00) |
| Students | 1.00 (0.76 – 1.32) | 0.96 (0.76 – 1.21) |
| Pensioners | 0.70 (0.56 – 0.88) | 0.61 (0.51 – 0.75) |
| Unemployed | 1.06 (0.84 – 1.35) | 0.82 (0.67 – 1.01) |
| Unknown | 1.14 (0.87 – 1.49) | 1.04 (0.83 – 1.31) |
| Multinomial model |  |  |
| IRI 12 (age, gender, SES, emg.) | (reference) | (reference) |
| IRI 13-24 (age, gender, SES, emg.) | 0.96 (0.86 – 1.06) | 0.82 (0.75 – 0.90) |
| IRI 25-36 (age, gender, SES, emg.) | 0.77 (0.69 – 0.86) | 0.75 (0.68 – 0.82) |
| IRI 37-60 (age, gender, SES, emg.) | 0.73 (0.61 – 0.88) | 0.93 (0.80 – 1.09) |
| Gender: woman | 0.86 (0.81 – 0.92) | 0.69 (0.65 – 0.73) |
| Age | 0.99 (0.99 – 1.00) | 0.98 (0.97 – 0.98) |
| SES: |  |  |
| Upper-level employees | 0.92 (0.74 – 1.15) | 0.74 (0.61 – 0.89) |
| Lower-level employees | 0.92 (0.74 – 1.14) | 0.69 (0.58 – 0.83) |
| Manual workers | 1.04 (0.82 – 1.30) | 0.84 (0.69 – 1.02) |
| Students | 0.99 (0.76 – 1.31) | 0.98 (0.78 – 1.24) |
| Pensioners | 0.71 (0.56 – 0.89) | 0.60 (0.50 – 0.73) |
| Unemployed | 1.05 (0.82 – 1.33) | 0.87 (0.71 – 1.07) |
| Unknown | 1.13 (0.86 – 1.47) | 1.10 (0.87 – 1.38) |
| Emergency appointment | 1.19 (1.12 – 1.27) | 0.52 (0.49 – 0.55) |
| Multinomial model |  |  |
| IRI 12 (age, gender, SES, CPI) | (reference) | (reference) |
| IRI 13-24 (age, gender, SES, CPI) | 0.94 (0.85 – 1.04) | 0.88 (0.81 – 0.97) |
| IRI 25-36 (age, gender, SES, CPI) | 0.74 (0.66 – 0.83) | 0.85 (0.77 – 0.94) |
| IRI 37-60 (age, gender, SES, CPI) | 0.69 (0.57 – 0.83) | 1.13 (0.96 – 1.31) |
| Gender: woman | 0.87 (0.81 – 0.93) | 0.69 (0.65 – 0.73) |
| Age | 1.00 (0.99 – 1.00) | 0.97 (0.97 – 0.97) |
| SES: |  |  |
| Upper- level employees | 0.91 (0.73 – 1.14) | 0.77 (0.64 – 0.93) |
| Lower -level employees | 0.91 (0.73 – 1.13) | 0.69 (0.58 – 0.83) |
| Manual workers | 1.04 (0.83 – 1.31) | 0.81 (0.67 – 0.99) |
| Students | 1.00 (0.76 – 1.31) | 0.95 (0.75 – 1.19) |
| Pensioners | 0.70 (0.56 – 0.88) | 0.60 (0.49 – 0.72) |
| Unemployed | 1.06 (0.84 – 1.35) | 0.81 (0.66 – 0.99) |
| Unknown | 1.14 (0.87 – 1.49) | 1.03 (0.82 – 1.29) |
| CPI max1 | 1.09 (0.91 – 1.31) | 0.88 (0.75 – 1.03) |
| CPI max2 | 1.13 (0.98 – 1.32) | 1.01 (0.89 – 1.14) |
| CPI max3 | 1.07 (0.91 – 1.26) | 1.05 (0.91 – 1.20) |
| CPI max4 | 1.24 (1.02 – 1.52) | 1.35 (1.13 – 1.60) |
| CPI maxX (edentulous) | 1.22 (0.65 – 2.30) | 6.03 (3.74 – 9.72) |
| Multinomial model |  |  |
| IRI 12 (age, gender, SES, DMFT) | (reference) | (reference) |
| IRI 13-24 (age, gender, SES, DMFT) | 0.94 (0.85 – 1.05) | 0.83 (0.75 – 0.90) |
| IRI 25-36 (age, gender, SES, DMFT) | 0.75 (0.67 – 0.84) | 0.76 (0.69 – 0.84) |
| IRI 37-60 (age, gender, SES, DMFT) | 0.69 (0.57 – 0.84) | 0.95 (0.81 – 1.11) |
| Gender: woman | 0.87 (0.81 – 0.93) | 0.68 (0.65 – 0.72) |
| Age | 1.00 (0.99 – 1.00) | 0.98 (0.98 – 0.99) |
| SES: |  |  |
| Upper-level employees | 0.91 (0.73 – 1.13) | 0.76 (0.63 – 0.91) |
| Lower-level employees | 0.91 (0.74 – 1.14) | 0.70 (0.58 – 0.84) |
| Manual workers | 1.04 (0.83 – 1.31) | 0.84 (0.70 – 1.03) |
| Students | 1.00 (0.76 – 1.31) | 0.95 (0.75 – 1.19) |
| Pensioners | 0.71 (0.56 – 0.89) | 0.59 (0.49 – 0.71) |
| Unemployed | 1.06 (0.84 – 1.35) | 0.85 (0.69 – 1.04) |
| Unknown | 1.14 (0.87 – 1.49) | 1.06 (0.84 – 1.33) |
| DMFT | 1.00 (0.99 – 1.00) | 0.97 (0.97 – 0.98) |
| Multinomial model |  |  |
| IRI 12 (age, gender, SES, chronic diseases) | (reference) | (reference) |
| IRI 13-24 (age, gender, SES, chronic diseases) | 0.94 (0.84 – 1.04) | 0.86 (0.78 – 0.94) |
| IRI 25-36 (age, gender, SES, chronic diseases) | 0.74 (0.66 – 0.83) | 0.82 (0.75 – 0.91) |
| IRI 37-60 (age, gender, SES, chronic diseases) | 0.69 (0.57 – 0.83) | 1.10 (0.94 – 1.28) |
| Gender: woman | 0.86 (0.81 – 0.92) | 0.68 (0.64 – 0.72) |
| Age | 1.00 (0.99 – 1.00) | 0.97 (0.97 – 0.97) |
| SES: |  |  |
| Upper-level employees | 0.91 (0.73 – 1.14) | 0.77 (0.64 – 0.93) |
| Lower-level employees | 0.92 (0.74 – 1.14) | 0.70 (0.58 – 0.84) |
| Manual workers | 1.05 (0.83 – 1.31) | 0.83 (0.68 – 1.00) |
| Students | 1.00 (0.76 – 1.32) | 0.97 (0.77 – 1.22) |
| Pensioners | 0.71 (0.57 – 0.90) | 0.66 (0.55 – 0.81) |
| Unemployed | 1.06 (0.84 – 1.35) | 0.83 (0.68 – 1.02) |
| Unknown | 1.14 (0.87 – 1.49) | 1.04 (0.83 – 1.31) |
| SK.103=Diabetes mellitus | 0.94 (0.81 – 1.10) | 0.86 (0.75 – 0.99) |
| SK.110=Parkinson’s disease and other comparable movement disorders | 1.81 (0.91 – 3.62) | 1.88 (0.97 – 3.62) |
| SK.112= Severe psychotic and other severe mental disorders | 0.97 (0.82 – 1.15) | 0.67 (0.57 – 0.78) |
| SK.201= Chronic cardiac insufficiency | 1.42 (0.92 – 2.19) | 1.32 (0.88 – 1.98) |
| SK.202= Disseminated connective tissue diseases, rheumatoid arthritis and comparable conditions | 0.91 (0.75 – 1.11) | 0.69 (0.58 – 0.83) |
| SK.203= Chronic asthma and similar chronic obstructive pulmonary diseases | 0.99 (0.86 – 1.14) | 0.85 (0.75 – 0.96) |
| SK.205= Chronic hypertension | 0.93 (0.83 – 1.05) | 0.83 (0.75 – 0.93) |
| SK.206= Chronic coronary heart disease and dyslipidaemia associated with chronic coronary heart disease | 0.99 (0.83 – 1.20) | 1.10 (0.93 – 1.30) |
| SK.207= Chronic arrhythmias | 0.83 (0.59 – 1.19) | 1.07 (0.79 – 1.46) |
| Multinomial model |  |  |
| IRI 12 (age, gender, SES, number of teeth) | (reference) | (reference) |
| IRI 13-24 (age, gender, SES, number of teeth) | 0.95 (0.85 – 1.05) | 0.90 (0.82 – 0.99) |
| IRI 25-36 (age, gender, SES, number of teeth) | 0.75 (0.67 – 0.84) | 0.88 (0.80 – 0.97) |
| IRI 37-60 (age, gender, SES, number of teeth) | 0.70 (0.58 – 0.84) | 1.18 (1.01 – 1.37) |
| Gender: woman | 0.86 (0.81 – 0.92) | 0.67 (0.63 – 0.71) |
| Age | 0.99 (0.99 – 1.00) | 0.97 (0.96 – 0.97) |
| Upper-level employees | 0.91 (0.73 – 1.14) | 0.77 (0.64 – 0.93) |
| Lower-level employees | 0.91 (0.73 – 1.14) | 0.69 (0.57 – 0.83) |
| Manual workers | 1.04 (0.83 – 1.30) | 0.80 (0.66 – 0.97) |
| Students | 1.00 (0.76 – 1.31) | 0.93 (0.74 – 1.18) |
| Pensioners | 0.69 (0.55 – 0.87) | 0.56 (0.46 – 0.67) |
| Unemployed | 1.06 (0.83 – 1.34) | 0.80 (0.65 – 0.98) |
| Unknown | 1.14 (0.87 – 1.49) | 1.00 (0.80 – 1.26) |
| Number of teeth | 0.99 (0.99 – 1.00) | 0.96 (0.96 – 0.97) |
| Multinomial model |  |  |
| IRI 12 (age, gender, SES, DT) | (reference) | (reference) |
| IRI 13-24 (age, gender, SES, DT) | 1.04 (0.93 – 1.15) | 1.03 (0.94 – 1.13) |
| IRI 25-36 (age, gender, SES, DT) | 0.86 (0.76 – 0.96) | 1.07 (0.97 – 1.19) |
| IRI 37-60 (age, gender, SES, DT) | 0.83 (0.68 – 1.00) | 1.51 (1.29 – 1.77) |
| Gender: woman | 0.90 (0.84 – 0.97) | 0.73 (0.69 – 0.77) |
| Age | 1.00 (0.99 – 1.00) | 0.97 (0.97 – 0.98) |
| SES |  |  |
| Upper-level employees | 0.92 (0.73 – 1.14) | 0.78 (0.64 – 0.94) |
| Lower-level employees | 0.90 (0.73 – 1.12) | 0.68 (0.57 – 0.82) |
| Manual workers | 1.00 (0.80 – 1.26) | 0.77 (0.64 – 0.94) |
| Students | 0.98 (0.74 – 1.28) | 0.92 (0.73 – 1.16) |
| Pensioners | 0.68 (0.54 – 0.85) | 0.58 (0.48 – 0.70) |
| Unemployed | 1.01 (0.80 – 1.28) | 0.76 (0.62 – 0.93) |
| Unknown | 1.08 (0.83 – 1.42) | 0.96 (0.76 – 1.20) |
| DT | 1.08 (1.06 – 1.10) | 1.12 (1.11 – 1.14) |
| Multinomial model |  |  |
| IRI 12 (age, gender, SES, emg., DT) | (reference) | (reference) |
| IRI 13-24 (age, gender, SES, emg., DT) | 1.05 (0.94 - 1.16) | 0.99 (0.90 - 1.09) |
| IRI 25-36 (age, gender, SES, emg., DT) | 0.88 (0.78 - 0.99) | 0.98 (0.88 - 1.08) |
| IRI 37-60 (age, gender, SES, emg., DT) | 0.86 (0.71 - 1.04) | 1.29 (1.10 - 1.51) |
| Gender: woman | 0.90 (0.84 - 0.96) | 0.74 (0.70 - 0.79) |
| Age | 0.99 (0.99 - 1.00) | 0.98 (0.98 - 0.98) |
| SES: |  |  |
| Upper-level employees | 0.93 (0.74 - 1.16) | 0.75 (0.62 - 0.91) |
| Lower-level employees | 0.91 (0.73 - 1.13) | 0.68 (0.56 - 0.82) |
| Manual workers | 1.00 (0.80 - 1.26) | 0.79 (0.65 - 0.96) |
| Students | 0.97 (0.74 - 1.28) | 0.94 (0.75 - 1.19) |
| Pensioners | 0.69 (0.55 - 0.86) | 0.56 (0.46 - 0.69) |
| Unemployed | 1.00 (0.79 - 1.27) | 0.80 (0.65 - 0.98) |
| Unknown | 1.07 (0.82 - 1.40) | 1.00 (0.79 - 1.26) |
| Emergency appointment | 1.17 (1.09 - 1.24) | 0.49 (0.47 - 0.52) |
| DT | 1.08 (1.06 - 1.10) | 1.14 (1.12 - 1.15) |
